# A Treatise on the Use of the Sympathetic Nerve and Its Ganglions; with Their Influence on Various Diseases of the Abdominal and Pelvic Viscera

**Published:** 1845-01

**Authors:** 


### A TREATISE ON THE USE OF THE SYMPATHETIC NERVE AND ITS GANGLIONS, WITH THEIR INFLUENCE ON VARIOUS DISEASES OF THE ABDOMINAL AND PELVIC VISCERA. By *T. Procter*, M.D. 4to. pp. 48. Highley, 1844.

WITHOUT going so far as to say he has completely made out his case, we think the author of this interesting essay has brought forward facts and opinions in reference to the functions and morbid affections of that portion of the nervous system, concerning which the most vague, unsatisfactory, and contradictory doctrines have hitherto prevailed, that must command attention and will stimulate observation by directing it into a more definite channel. That additional and multiplied researches will be required before what he has advanced can take the position of an assured fact, he does not endeavour to conceal; and, having made what must at the very least be termed a most happy suggestion, he contents himself with pointing out a few corroborations which the phenomena of disease and the action of remedies seem to supply. If more extended observation confirms the views set forth, the practical consequences will ere long exhibit themselves abundantly enough.

The contemplation of the intimate anatomical relations of the sympathetic nerve with the arterial system, induces Dr. Procter to conclude that its office consists in regulating the contractility of the blood-vessels. Nerves derived from other sources frequently accompany arteries for a long portion of their course, never, however, transmitting branches large or small to them, so that their juxta-position must be referred to some other cause than for affording facility for influencing their movements. The sympathetic, on the contrary, is emphatically the nerve of these vessels, enmeshing and penetrating them on every side, and following their minutest ramifications to their ultimate distribution. After describing the anatomical relations of the nerve Dr. Procter thus proceeds :—

"The nearest approach to a positive determination of its use that we can come to with our present limited knowledge is, that it is for the purpose of regulating the tonic contraction of the arterial system, and for *nothing else;* however, it is difficult to expound or afford the requisite proofs of this opinion, nor am I aware that public attention has at all been called to it. I venture, therefore, allowing this idea to form the basis of my investigation, to proceed to explain my present views upon the subject, first calling the reader's attention to the remarkable fact with which the discoveries of Sir C. Bell have made us acquainted—that there is not a part of the human body that is not supplied with two or three sets of nerves according to the simplicity or the intricacy of its functions: the excitors for sensibility, the motors for movement, and the respiratory system for the complicated purposes of respiration. When we see, by the discoveries of Dr. M. Hall, that we have a presiding and regulating power over all the sphincters and muscles of the body through the medulla oblongata and the medulla spinalis, and, in fact, that there is scarcely an organ in the human body that is not now *known to have* a moving and directing power; is it then probable, or even possible, that so important a system as the arterial should be without such a controlling and directing power? Acknowledging that it is not, as every one must necessarily do, and coupling this with the fact that there is a large and evidently important system of nerves exclusively surrounding, embracing, and running into the coats of the arterial system, of which we know little or nothing; and when we see the remarkable way in which anatomy bears out this opinion, I would venture to predict that so surely as anatomy led Sir C. Bell on step by step to his admirable, lucid, and conclusive arrangement of the other nervous systems, so surely does anatomy point out to us most distinctly the functions of *this* nervous system; and doubtless the time will arrive when it will be capable of demonstration: difficult as I confess it now appears to be, from its peculiar situation in the body, and from its apparent total want of functional connection with the other systems. It is singular that up to this period no author has sufficiently pointed out the remarkable difference in appearance and structure between the ganglia of the sympathetic and those of the spinal nerves. A single glance will be sufficient to shew this very marked difference. It is seen in the sympathetic ganglion that the nerves appear to be more like elongations of the ganglion, each coming out clear and distinct, like so many tails, the ganglion itself being of an oblong shape and smooth. In the spinal ganglia the nerves are seen entering the globe-like body of a ganglion in bundles, leaving it in the same divided or fascicular form."

After alluding to the proofs derived from the experiments of Philip, Flourens, and others, of the non-dependence of the circulation upon the cerebro-spinal system, the author, in another part of his work, thus expresses himself.

" It is self-evident, then, that it is to the sympathetic (and that alone) that we must look for regulating the arterial system. And it will be observed that in all parts of the animal body where large and sudden supplies of blood are required, such as the heart, stomach, bowels, and organs of generation, we have the ganglionic or sympathetic system very fully developed, and, as far as I can judge, in ratio to the amount of blood supplied to the several organs: on the contrary, in some parts of the body, and in the extremities where the flow of blood is more regular and not subjected to those *sudden calls for large supplies of blood at irregular periods,* we find this nerve manifestly decreasing in size: and, indeed, as far as we can judge with the naked eye, ceasing altogether in some parts. Still I perfectly agree with Sir Charles Bell that it is distributed all over the body: but whether its influence is confined to regulating the small arteries which supply the coats of the vessels, or whether the same influence is continued by it over the whole circulating medium of the extremities and other parts that it manifestly has over the abdominal viscera, must, I fear, be left to a more enlarged enquiry."

An experiment is related which consisted in exposing the branch of the sympathetic nerve joining the ischiatic, and one of the arteries of the leg in a horse that had been killed by division of the medulla spinalis, and then connecting the nerve with the positive and the artery with the negative pole of a galvanic battery. Pulsation in the artery was not only induced, but circulation in the minuter vessels was also excited. Similar experiments afterwards repeated gave corresponding results.

For anticipatory replies to some of the objections which may be raised to this explanation of the function of the nerve, we must refer to the work itself; and can only briefly advert to the practical and therapeutical applications. These, upon the very threshold of the inquiry, are of course few and somewhat conjectural; but, the true physiology and pathology of the nerve once established, they will multiply rapidly in number and certitude.

"If debility of its fine threads be a sufficient cause to throw into dissonant action the heart and arteries in some cases, should we not be inclined to abandon the practice which rests on depletion, counter-irritation, and treatment of this kind, and substitute in their place remedies and a regimen which may fortify and soothe the nerve? If dropsy be often caused by obstruction in the flow of blood through the portal system, and this debility of action in the portal veins depend upon an exhausted state of the sympathetic, shall we torment the patient, and perhaps increase the debility in question, by the use of hydragogues and diuretic medicine, which can only be intended to act on the *effect* of the original malady, leaving the *cause* unacted upon (at least beneficially so); or, finally, knowing the inevitable tendency of the disorder of this nerve to engender serious organic maladies, shall we not watch for the first symptoms of its decline to restore its tone, or at all events retard the period of its loss of energy? But it will be asked, where are the means of restoring to a nerve the power it has lost? It may be replied, that although the restoration of a function utterly lost by time or long stimulation may be impossible, yet there is abundant evidence to shew, that other nerves have their functions restored in a direct mode by medicine. Take, for example, the agency of strychnine on paralysis of the spinal nerves, of carbonate of iron on neuralgia, and what evidence have we that all the beneficial effects of stimulants and tonics are not through the influence of this nerve?

"Nor can any one be ignorant that a regimen in which regularity and rest from mental emotions or exertions are insisted upon, has produced still more remarkable restorations. I do not doubt that many with myself will think that by similar means the impeded functions of the sympathetic have been restored.

"A very curious fact confirmatory of the views which I have taken upon this subject, is, I think, afforded by the remarkable analogy which will be seen to exist between the action of strychnia and of electric or galvanic influence upon functional disorders in some of the organs over which the sympathetic, and *that alone*, has control; not only as regards the kind of power acting upon the nerves, but also its influence on this particular system of nerves."

The outlines of several cases are given as confirmatory of the author's opinion of the *modus operandi* of strychnia; viz. that it influences the digestive, uterine, generative organs, &c. through the medium of the sympathetic, upon which it exerts a powerful direct action. Among these are examples of *impotence, amenorrhœa,* and *spasmodic asthma combined with dyspepsia.* In nearly all, the effects of the medicine were rapid and decided in restoring the tone of the defective organ. In two of the cases of amenorrhœa which are related, galvanism acted in an equally beneficial manner. "It is by no means an unfrequent occurrence for menstruation to take place while the patient is being operated upon by galvanism; the same thing also occurs while under the influence of a single dose of strychnia."

A friend, Dr. Price, writing to the author concerning his experience in the use of strychnia in *habitual constipation,* after detailing a case of its beneficial influence, observes—

"I beg to add that I have perceived the same effects in very many other instances, where the bowels have been induced to perform their functions daily, and these effects have continued for a longer or shorter period, requiring afterwards, in some instances, to take a little aperient medicine, as would be the case with persons not habitually costive. In a case, a few days ago, of a female whose bowels had been at all times constipated, I was compelled to repeat the strychnia. About four or five months before she had been very effectually relieved by this medicine: the necessity for this visit arose, by her own admission, from inattention to natural calls, and other circumstances. I should remark here that she has been married for several years, but she has never been pregant: she never menstruated freely till after taking the strychnia. This is also a very frequent good effect of the administration of this medicine."

Even many of those who may not feel disposed to agree with the author as to the manner in which strychnia effects these beneficial changes in a class of affections often rebellious, will gladly avail themselves of the information he furnishes of its curative agency. Three plates illustrate some of the peculiarities of the distribution of the sympathetic.

